# New Reduction Technique for Traumatic Posterior Glenohumeral Joint Dislocations

**DOI:** 10.5811/cpcem.2019.11.44790

**Published:** 2020-01-24

**Authors:** Morteza Khodaee

**Affiliations:** University of Colorado School of Medicine, Department of Family Medicine and Orthopedics, Division of Sports Medicine, Denver, Colorado

## Abstract

Traumatic posterior glenohumeral joint (GHJ) dislocation is a rare condition which can be missed if it is not suspected. Clinical presentation may be subtle, but limitation in range of motion in patient with acute trauma should warrant obtaining a thorough history, performing a comprehensive physical examination, and acquiring at least a 3-view plain radiography. Reduction can be achieved with a direct pressure to the posterior aspect of the humeral head.

## CASE PRESENTATION

**Image 1:**
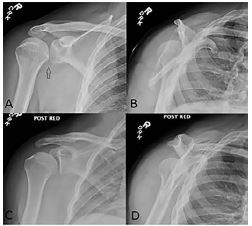
Right shoulder plain radiography reveals posterior glenohumeral dislocation (arrow) on AP (A) and scapular Y (B) views. Post-reduction Grashey (C) and scapular Y (D) views confirmed the reduction. No fracture is present on these images.

**Image 2:**
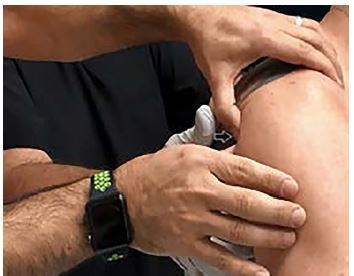
Reduction of the posterior glenohumeral dislocation with anteriorly directed pressure to the posterior humeral head (arrow).

A 44-year-old male presented at a ski clinic shortly after a fall directly onto his right shoulder while skiing. He was unable to move his right arm due to pain. His past medical history was significant for right shoulder dislocation about 25 years earlier. He did not recall the type of dislocation he suffered at that time. He did not undergo any surgeries. He had an active lifestyle and denied experiencing any other shoulder injuries since then. On physical examination, he was holding his right arm in an adducted and internally rotated position. He had a subtle deformity (sulcus sign) in his right shoulder. The patient was unable to tolerate a passive abduction due to the pain. His neurovascular examination was normal. Plain radiography revealed posterior glenohumeral joint (GHJ) dislocation (Image 1). We reduced his posterior GHJ dislocation using direct pressure to the posterior aspect of his humeral head in a sitting position without any analgesics (Image 2 and Video). Reduction was confirmed by post-reduction radiographes (Image 1).

## DISCUSSION

Most posterior GHJ dislocations are atraumatic (e.g., seizures and electrocutions).^1^,^2^ Traumatic posterior GHJ dislocations account for less than 1% of all GHJ dislocations.^2^,^3^ Due to subtle clinical presentations, particularly among the elderly with atraumatic etiology, and subtle signs on plain radiography, up to 80% of these dislocations are misdiagnosed for months.^1^–^3^ If the dislocation is not obvious on AP, AP oblique (Grashey), and lateral (scapular Y) radiography views, an axillary (or at least a Velpeau axillary) view should be obtained.^2^–^4^ As the majority of patients with posterior GHJ dislocation are diagnosed late, reduction usually requires conscious sedation or general anesthesia.^1^–^3^

There is a gap in the literature on the management of acute traumatic posterior GHJ dislocations.^2^ ^3^ Similar or modified reduction techniques for anterior GHJ dislocation are recommended for acute traumatic posterior GHJ.^1^–^4^ Most of these recommended techniques involve longitudinal traction, forward flexion, and internal and external rotation of the shoulder.^1^–^5^ The humeral head is more palpable and more superficial in patients with posterior GHJ dislocations compared to patients with anterior GHJ dislocations. It seems that significant anteriorly directed force can be transferred to the humeral head by applying direct pressure in patients with posterior GHJ dislocations. To the best of our knowledge, this is the first time that direct posterior pressure to the humeral head is applied alone. However, anteriorly directed pressure to the humeral head in conjunction with longitudinal tractions, and internal and external rotations have been recommended previously.^1^,^2^,^5^ This technique requires minimum patient cooperation. Large studies are required to evaluate the effectiveness of this technique for patients with acute traumatic posterior GHJ dislocations.

**CPC-EM Capsule**

What do we already know about this clinical entity?

Traumatic posterior glenohumeral joint (GHJ) dislocation is rare. Unless clinically suspected, there may be a significant delay in diagnosis and management of a traumatic posterior GHJ dislocation.

What is the major impact of the image(s)?

The image and the video show how you can attempt this new technique.

How might this improve emergency medicine practice?

The new proposed technique may improve the reduction procedure by decreasing the numbers of the attempts and eliminating the need for pre-procedural sedation.

## Supplementary Information

Video. Reduction of the posterior glenohumeral dislocation with anteriorly directed pressure to the posterior humeral head (arrow).
